# Synaptic mechanisms underlying the intense firing of neocortical layer 5B pyramidal neurons in response to cortico-cortical inputs

**DOI:** 10.1007/s00429-019-01842-8

**Published:** 2019-02-12

**Authors:** Alejandro Sempere-Ferràndez, Salvador Martínez, Emilio Geijo-Barrientos

**Affiliations:** 1 Instituto de Neurociencias, Universidad Miguel Hernández, Consejo Superior de Investigaciones Científicas, Campus de San Juan, Avenida Ramón y Cajal s/n, San Juan de Alicante, 03550 Alicante, Spain; 2 CIBERSAM (Centro de Investigación en Red en Salud Mental), Madrid, Spain

**Keywords:** Thick-tufted pyramidal neuron, Fast-spiking interneuron, Retrosplenial cortex

## Abstract

In the neocortex, large layer 5B pyramidal neurons implement a high-density firing code. In contrast, other subtypes of pyramidal neurons, including those in layer 2/3, are functionally characterized by their sparse firing rate. Here, we investigate the synaptic basis of this behavior by comparing the properties of the postsynaptic responses evoked by cortical inputs in layer 5B and layer 2/3 pyramidal neurons in vitro. We demonstrate that a major determinant of the larger responsiveness of layer 5B with respect to layer 2/3 pyramidal neurons is the different properties in their inhibitory postsynaptic currents (IPSCs): layer 5B pyramidal neurons have IPSCs of lower amplitude and the temporal delay between the excitatory and inhibitory synaptic components is also larger in these cells. Our data also suggest that this difference depends on the lower gain of the cortical response of layer 5 parvalbumin-positive fast-spiking (PV-FS) interneurons with respect to PV-FS cells from layer 2/3. We propose that, while superficial PV-FS interneurons are well suited to provide a powerful feed-forward inhibitory control of pyramidal neuron responses, layer 5 PV-FS interneurons are mainly engaged in a feedback inhibitory loop and only after a substantial recruitment of surrounding pyramidal cells do they respond to an external input.

**Electronic supplementary material:**

The online version of this article (10.1007/s00429-019-01842-8) contains supplementary material, which is available to authorized users.

## Introduction

A landmark of neocortical organization is its laminar arrangement (Lewis 1880), with neurons from different layers showing distinct genetic, morphologic and functional properties (Thomson and Lamy 2007). Electrophysiological recordings and calcium imaging in sensory, motor, and associative cortex in vivo have revealed that pyramidal neurons in layer 2/3 implement a sparse firing code (Beloozerova et al. 2003a, b; Crochet and Petersen 2006; Sakata and Harris 2009; Sawinski et al. 2009; Crochet et al. 2011). These neurons remain silent or are sharply tuned, firing in response to specific configurations of the stimuli. In contrast, large pyramidal neurons in layer 5B with thick apical dendritic tufts are broadly tuned and respond in a more unspecific manner (Manns et al. 2004; Sun et al. 2013; Lur et al. 2016). The different activity patterns shown by layer 2/3 and layer 5B pyramidal neurons are observed both in anesthetized (de Kock et al. 2007) and awake animals (Greenberg et al. 2008; de Kock and Sakmann 2009), indicating that this laminar distinction is a general signature of cortical function.

Electrophysiological recordings in vitro have shown that in response to local inputs, layer 5 cells have a larger excitation to inhibition (E/I) balance than those in layer 2/3 (Adesnik and Scanziani 2010). Several studies indicate that superficial microcircuits are characterized by a potent feed-forward inhibitory input arising from PV-FS cells (Holmgren et al. 2003; Mateo et al. 2011; Avermann et al. 2012), which could explain the lower E/I balance and sparse recruitment of layer 2/3 pyramidal neurons. However, fewer reports have addressed the role of PV-FS inhibitory neurons in deeper layers, and a direct comparison with those in superficial layers is missing.

To get insights into the different coding strategies of both pyramidal subpopulations, and to test the hypothesis that the particularities of the inhibitory subnetworks of superficial and deep layers could underlay such difference, we have compared the synaptic properties of the responses evoked by cortical inputs in layer 2/3 and layer 5B large pyramidal neurons. In addition, we have also studied the integration of cortical inputs in superficial and deep PV-FS interneurons. For this, we have used a slice preparation with conserved transhemispheric connectivity.

In our conditions, single-pulse stimulation of the upper layers of the cortex resulted in a larger recruitment of contralateral pyramidal neurons in layer 5B than in layer 2/3. This depended on a larger excitatory/inhibitory balance of the synaptic response of layer 5B pyramidal neurons, which in turn was explained by larger EPSCs and smaller and delayed IPSCs. Our data points to different laminar dynamics of PV-FS-dependent inhibition as a main contributor of the lower gain of the inhibitory response and larger responsiveness of layer 5B pyramidal neurons; while superficial PV-FS interneurons were strongly recruited with a lower stimulation threshold than surrounding pyramidal cells, in layer 5 the responses of PV-FS cells were smaller and the stimulus threshold to evoke spikes was higher than in pyramidal neurons. We propose that while PV-FS interneurons in upper layers provide a potent feed-forward control of pyramidal responsiveness in response to incoming excitation, in layer 5 PV-FS interneurons require the amplifying signal of surrounding large pyramidal cells; thus, in layer 5, PV-FS inhibition is characterized by feedback dynamics.

## Methods

### Animals

For recordings of pyramidal neurons, brain slices of neocortex were prepared from mice of either sex (C57-BL6 strand; 17–21 postnatal days). For recordings of parvalbumin-expressing fast-spiking interneurons (PV-FS), we used the Pvalb-Cre;RCE mouse in which PV-expressing neurons are labeled with EGFP; these animals were made by crossing Palvb-Cre mice (Hippenmeyer et al. 2005) with a Cre-dependent EGFP reporter line RCE;FRT (Sousa et al. 2009).

### Slice preparation

Animals were killed by cervical dislocation and their brains were quickly excised and submerged in ice-cold low-Ca^2+^/high-Mg^2+^ cutting solution (composition in mM: NaCl 124, KCl 2.5, NaHCO_3_ 26, CaCl_2_ 0.5, MgCl_2_ 2, NaH_2_PO_4_ 1.25, glucose 10; pH 7.4 when saturated with 95% O_2_ + 5% CO_2_). Coronal slices (350 µm thick) were cut using a vibratome (Leica VT-1000; Germany) and transferred to a glass beaker, in which the tissue was submerged in artificial cerebrospinal fluid (ACSF; composition in mM: NaCl 124, KCl 2.5, NaHCO_3_ 26, CaCl_2_ 2, MgCl_2_ 1, NaH_2_PO_4_ 1.25, glucose 10; pH 7.4 when saturated with 95% O_2_ + 5% CO_2_) at 34 °C for 30 min. 2–3 slices per brain including the anterior part of the retrosplenial cortex were selected (anteroposterior axis − 1.4 to − 2.5 mm from Bregma). Selected slices were stored submerged in ACSF for at least 1 hour at room temperature before recordings were made. One slice at a time was transferred to a submersion-type recording chamber and kept at 32–34 °C during the recording period. The ACSF used to bath the slices was fed into the recording chamber at a rate of 2–3 ml min^−1^ and was continuously bubbled with a gas mixture of 95% O_2_ + 5% CO_2_.

### Intracellular recordings

Somatic whole-cell recordings were made under visual control using an upright microscope (Olympus BX50WI) equipped with Nomarski optics and a water immersion lens (40x) in the retrosplenial agranular region (0–0.75 mm from midline). Recordings were obtained in current-clamp or voltage-clamp mode with a patch-clamp amplifier (Multiclamp 700B, Molecular Devices, USA). No correction was made for the pipette junction potential (which was estimated to be about − 10 mV using the junction potential calculator included in the pClamp software). Voltage and current signals were filtered at 2–4 kHz and digitized at 20 kHz with a 16-bit resolution analog to digital converter (Digidata 1322A, Axon Instruments). The generation and acquisition of pulses were controlled by pClamp 9.2 software (Axon Instruments). Patch pipettes were made from borosilicate glass (1.5 mm o.d., 0.86 mm i.d., with inner filament) and had a resistance of 4–7 MΩ when positive pressure was applied.

Current-clamp experiments were performed with an intracellular solution containing (in mM): 130 K-gluconate, 5 KCl, 5 NaCl, 5 EGTA, 10 HEPES, 2 Mg–ATP, 0.2 Na–GTP, 0.01 Alexa Fluor 594; pH 7.2, adjusted with KOH; 285–295 mOsm. Voltage-clamp recordings were performed with an intracellular solution containing (in mM): 135 Cs-methanesulfonate, 10 NaCl, 5 EGTA, 10 HEPES, 2 Mg–ATP, 0.2 Na–GTP, 0.01 Alexa Fluor 594; pH 7.2, adjusted with CsOH; 285–295 mOsm. The theoretic Nernst equilibrium potentials for the K-based internal solution were (in mV) *E*_K_ = − 105.7, *E*_Na_ = 89.3, *E*_Cl_ = − 68.5). The theoretic Nernst equilibrium potentials for the Cs-based internal solution were (in mV) *E*_Na_ = 71.4, *E*_Cl_ = − 68.5.

Current-clamp recordings were performed at the resting membrane potential of the neuron. Series resistance (*R*_s_) was measured and balanced on-line under visual inspection assisted by the Bridge Balance tool of Clampex software. *R*_s_ was monitored at the beginning and at the end of each protocol, and re-balanced if needed. Neurons in which *R*_s_ > 40 MΩ were discarded (typical *R*_s_ 10–25 MΩ).

For voltage-clamp experiments, EPSCs and IPSCs were recorded with holding potentials of − 70 and 0 mV, the measured reversal potential for the inhibitory and excitatory synaptic currents, respectively. Neurons in which *R*_s_ > 30 MΩ were discarded (typical *R*_s_ was between 10 and 25 MΩ). The error in the measure of the membrane potential (*V*_e_) was computed as *V*_e_ = *I*_hold_ × *R*_s_, where *I*_hold_ was the holding current needed to set the holding potential (*V*_hold_). To hold the neuron at the desired membrane potential (*V*_m_), we set *V*_hold_ = *V*_m_ + *V*_e_. Quantification of intrinsic membrane properties and synaptic responses was performed on Clampfit10.3.

### Stimulation of cortical slices

Slices were stimulated with a concentric bipolar electrode (CBAFC75, Frederick Haer & Co, USA) placed on layer 2/3 of the homotopic contralateral cortex with respect to the recording region. Single pulses (stimulus intensity 30–800 µA, 0.1 ms) were applied at a frequency of 0.2 Hz. Integrity of the projection was assessed by extracellular recordings prior to intracellular experiments. In our conditions, direct postsynaptic responses were evoked in contralateral neurons by the recruitment of pyramidal neurons ipsilateral to the stimulus. However, excitatory synaptic responses can also arise from the spiking activity evoked in pyramidal neurons ipsilateral to the recording site. At low-intensity stimulus (about 100 µA or less) responses are exclusively evoked by callosal axons while at higher stimulus intensities (200–500 µA), despite callosal inputs remaining dominant, local pyramidal neurons from layer 2/3 and layer 5B are also recruited. Quantification of this contribution can be found in a previous report (Sempere-Ferràndez et al. 2018).

For recordings of putative unitary (single axon) responses, a minimal stimulation protocol was applied. This method consisted in setting the stimulus intensity at the minimal amplitude at which a synaptic response is evoked. In a typical experiment, initial stimulus intensity was set to a subthreshold level (success rate = 0) and then it was progressively increased in small steps (5 µA or less) until a response appeared, usually with a low success rate < 0.7. Exclusion of possible compound responses was performed off-line under visual inspection; compound responses were identified by the presence of discontinuities in the rising phase or changes in the peak amplitude of the response. A high jitter (> 0.4 milliseconds) was also considered as an exclusion criterion for putative unitary EPSCs, but not for putative unitary IPSCs, whose disynaptic nature increases their temporal variability.

### Identification of cortical neuron subtypes

During recordings, identification of layer 2/3 pyramidal neurons and layer 5B large pyramidal neurons was done according to previously described criteria (Sempere-Ferràndez et al. 2018). Recorded cells were considered as pyramidal neurons only if they showed dendritic spines and a dominant apical dendritic tree extensively ramifying in layer 1 after intracellular staining with AlexaFluor 594 and/or biocytin (see examples in supplementary Fig. 1a and b). In layer 2/3, we recorded only pyramidal neurons whose somas were placed below the border between layer 1/2 and at not more than 300 µm from the pial surface. In addition, these neurons responded to suprathreshold current steps with a regular spiking pattern (supplementary Fig. 1c). Our sample of layer 5B pyramidal neurons only included pyramidal neurons with large somas (> 200 µm^2^, as seen in the living slice under DIC optics) located in the upper part of layer 5B (between ~ 400 and ~ 550 µm from pia) and that had a membrane input resistance < 80 MΩ. From now on, we will use the term “L2/3” to refer the pyramidal neurons of layer 2/3 and the term “L5BL” to refer the large pyramidal neurons of upper layer 5B. We have previously shown that in the retrosplenial cortex, large pyramidal cells of layer 5 correspond to pyramidal neurons with thick apical dendritic tufts branching in layer 1 and that the subset of these neurons located in the upper part of layer 5B respond with larger synaptic potential and fire more frequently in response to contralateral stimuli that neurons locate in the lower part of layer 5B (Sempere-Ferràndez et al. 2018); for this reason, we have focused our study in the L5BL neurons of the upper part of layer 5B. Layer 5B also contains pyramidal neurons with medium-size somas (< 200 µm^2^), a higher input resistance (> 100 MΩ) and poorly developed apical dendritic tufts in layer 1 (Sempere-Ferràndez et al. 2018). We have excluded these neurons from this study as they show a low responsiveness to the stimulation protocol implemented here (Sempere-Ferràndez et al. 2018). For data regarding the intrinsic electrophysiology of layer 2/3 and layer 5BL pyramidal neurons included in this study, see supplementary Fig. 1.

### Statistics

For comparisons of non-paired data, the two-tailed Mann–Whitney rank sum test was employed. For pairs of neurons recorded sequentially (in this case, the position of the stimulus electrode and the intensities were the same for both neurons), the two-tailed Wilcoxon signed rank test was employed. For comparison of proportions, the two-tailed *Z* score test was employed. Statistical analysis was performed in OriginPro8 (Origin Lab Corporation) or Sigma Stat 3.11 (Systat Software Inc.).

## Results

We have studied the synaptic determinants of the responses of layer 2/3 and layer 5B pyramidal neurons in response to cortical inputs in slices of mice of 17–21 postnatal days.

### Physiological properties of cortico-cortical synapses in L2/3 and L5BL pyramidal neurons

**Fig. 1:**
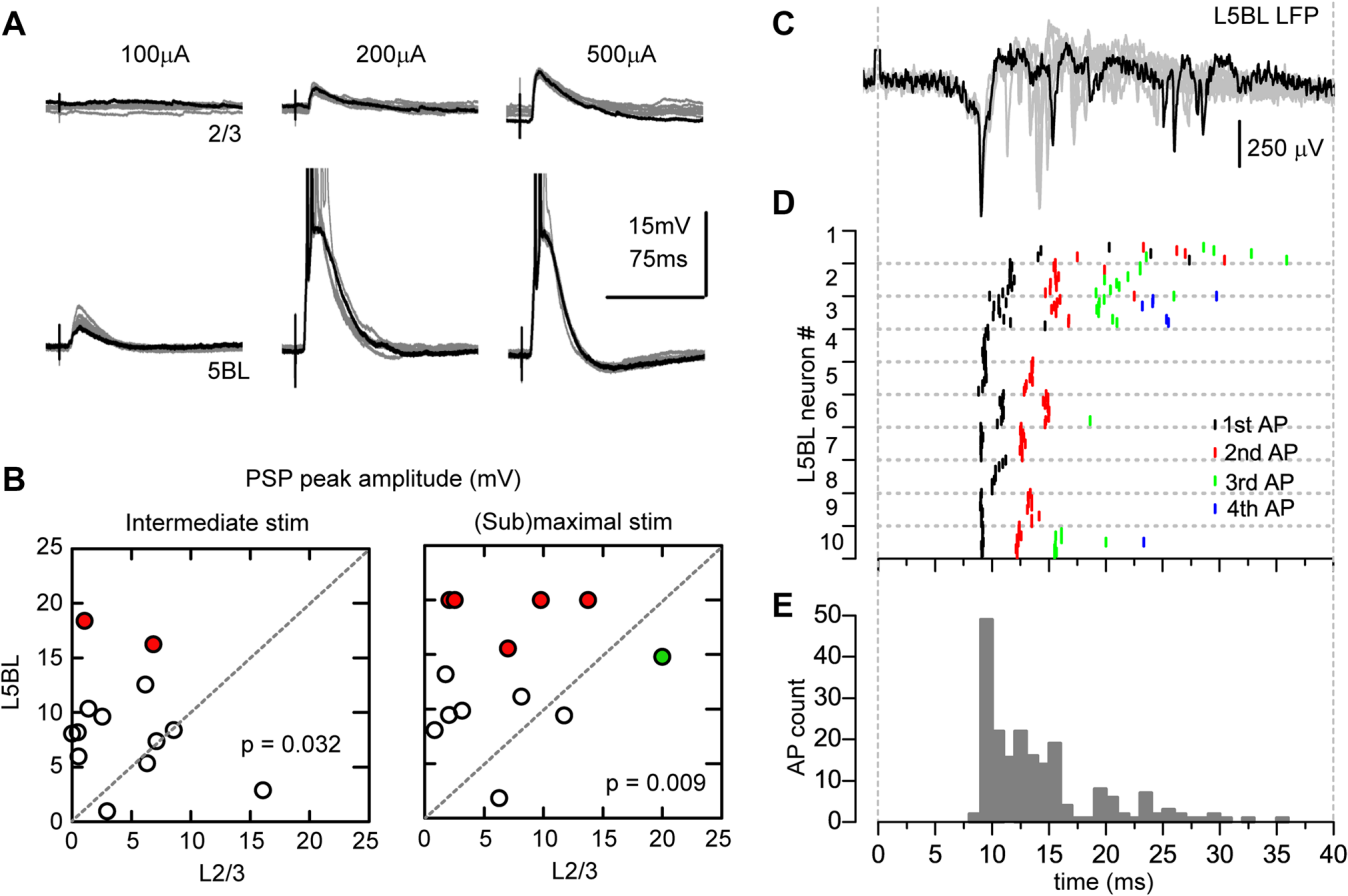
Larger gain of the synaptic response of L5BL vs L2/3 pyramidal neurons. **a** Postsynaptic potentials evoked in a L2/3 (upper panel) and a L5BL pyramidal neuron (lower panel) recorded sequentially in the same slice in response to electrical stimulation of the contralateral cortex. 10 superimposed responses are shown for each neuron at each stimulus intensity (100–500 µA); one trace is highlighted in black. Action potentials in the L5BL pyramidal neuron are truncated. **b** PSP peak amplitude in a sample of L2/3 and L5BL pyramidal neurons sequentially recorded (*n* = 13 pairs) in response to intermediate (left; stimulus intensity: 221 ± 34 µA) and near-maximal (right; stimulus intensity: 485 ± 28 µA) stimulus intensities. Each symbol represents the average PSP amplitude of ten consecutive responses. Red circles represent L5BL neurons in which firing was evoked at least in 5/10 responses; green symbol shows the L2/3 neuron that fired in response to near-maximal stimulus. In neurons in which firing was evoked by the stimulus, a value of 20 mV was applied for statistical comparison and graphical representation. The average amplitudes of the PSPs were 4.5 ± 1.2 mV (L2/3 neurons, intermediate stimuli), 6.6 ± 1.5 (L2/3 neurons, near-maximal stimuli), 8.6 ± 1.4 (L5BL neurons, intermediate stimuli) and 13.5 ± 5.6 (L5BL neurons, near-maximal stimuli). The difference in peak amplitude was statistically significant (intermediate stimuli: *p* = 0.032; near-maximal stimuli: *p* = 0.009). **c** Extracellular recording of the local field potential evoked in layer 5B in response to contralateral stimulation. Notice the abundant presence of fast, negative deflections, presumably as a result of the firing activity of layer 5 neurons. 10 superimposed responses are shown, one highlighted in black. **d** Latency of the action potentials in the subsample of L5BL pyramidal neurons recruited by submaximal stimulation (*n* = 204 spikes from 10 L5BL pyramidal neurons, 6 slices from 6 mice, stimulus intensity 450 ± 25 µA). For each neuron, action potential latency is represented for each one of the ten consecutive responses. **e** Frequency histogram of the latency of the action potential shown in **d**. Notice that in agreement with the extracellular recording (G), there is a peak before 10 ms and a long tail that expands until 40 ms after the stimulus. Temporal scales in **c–e** are the same

First, we recorded the postsynaptic potentials (PSPs) evoked by stimulation of the superficial layers of the retrosplenial cortex in contralateral L2/3 and L5BL pyramidal neurons (see “Methods” for details). PSPs were larger and induced firing more frequently in L5BL compared to L2/3 pyramidal neurons (Fig. 1a, b); this result is consistent with and extends our previous observations showing that the PSPs evoked by contralateral stimulation tend to be larger in layer 5B large pyramidal neurons (particularly in those whose somas are placed in the upper part of layer 5B) than in layer 2/3 pyramidal neurons (Sempere-Ferràndez et al. 2018). In Fig. 1a, we show the PSPs recorded in a pair of neurons formed by a L2/3 and a L5BL pyramidal cell in the same slice. In this example, synaptic responses were evoked in response to current pulses of 100, 200, and 500 µA. The PSPs were larger in the L5BL pyramidal cell, which fired a burst of 2–4 action potentials in response to 200 and 500 µA, while in the L2/3 pyramidal neuron the PSPs were subthreshold for the three intensities tested.

To standardize the stimulus intensities used in different slices and experiments, we used two intensities: 4× and 8× the threshold value found for the first neuron recorded in a pair. Across different slices, the 4× level was, on average, close to 200 µA and the 10× level was close to 500 µA and, therefore, we will refer to stimulus intensities of about 200 µA as “intermediate” and intensities about 500 µA as “near-maximal”. In response to intermediate intensities, the PSPs reached the action potential (AP) threshold in 0 out of 13 L2/3 and 2 out of 13 L5BL pyramidal neurons (*p* = 0.146 and *p* = 0.007 respectively). With near-maximal stimulation, 1 out of 13 L2/3 and 5 out of 13 L5BL pyramidal neurons fired in response to the stimuli (*p* = 0.063). In our whole sample of recordings, which included non-paired recordings of L2/3 and L5BL (*n* = 28 L2/3 and 21 L5BL pyramidal neurons), the ratio of neurons recruited by the stimuli was significantly larger among L5BL pyramidal cells at both stimulus intensities tested: in response to intermediate intensities, 0 out of 28 L2/3 and 5 out of 21 L5BL pyramidal neurons were recruited (*p* = 0.007) and with near-maximal stimulation 2 out of 28 L2/3 and 10 out of 21 L5BL pyramidal neurons fired in response to the stimuli (*p* = 0.001).

Those L5BL pyramidal neurons that fired in response to contralateral stimuli typically responded with a burst of 2–4 action potentials on top of a large PSP (in a sample of 10 firing L5BL pyramidal neurons, only 2 responded with just one action potential in response to near-maximal stimulation). The latency of the first spike was usually below 10 ms after the stimulus onset (Fig. 1c–e; median latency 9.48 ms, range 9.03–20.00 ms), and showed a low jitter across several trials in a neuron (Fig. 1d). The following spikes in a burst appeared in a time window that lasted about 40 ms after the stimulus and showed a larger latency variability across trials and neurons (Fig. 1d, e). Overall, these data indicated that, in spite of their significantly larger rheobase (supplementary Fig. 1), L5BL pyramidal neurons were more excitable by cortical inputs than L2/3 pyramidal cells.

**Fig. 2:**
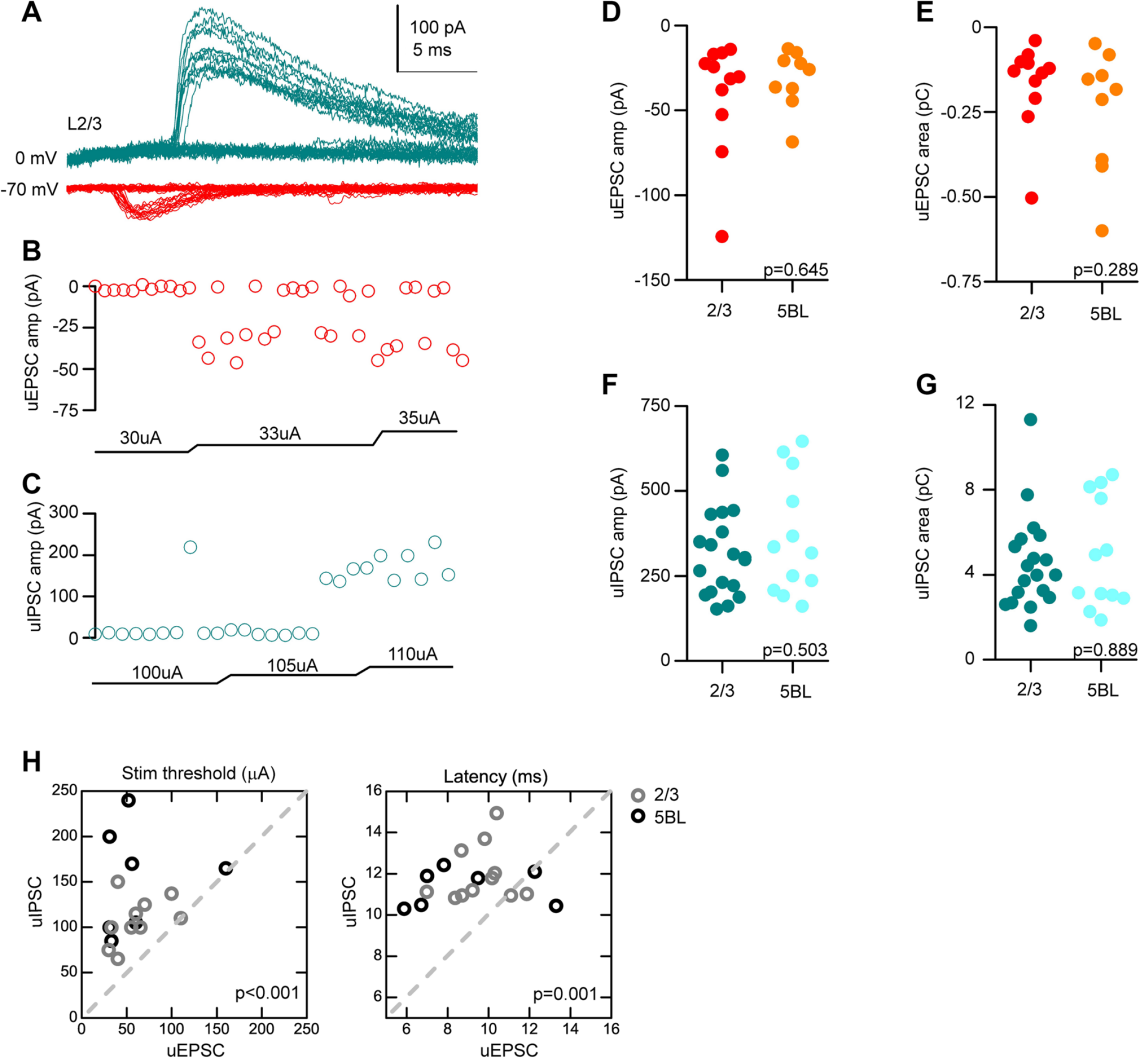
Properties of unitary EPSCs and IPSCs in L2/3 and L5BL pyramidal neurons. **a** Example of a putative unitary EPSC (red) and IPSC (blue) recorded in a L2/3 pyramidal neuron in response to a minimal stimulation protocol. Notice the abundant presence of failures and the longer latency of the unitary IPSC. **b, c** Peak amplitude and stimulus intensity employed for the unitary EPSCs (**b**) and IPSCs (**c**) shown in **a**. Notice that uEPSCs had a lower stimulation threshold than uIPSCs. **d, e** Peak amplitude and area of the uEPSCs recorded in a sample of L2/3 and L5BL pyramidal neurons (*n* = 11 and 9, respectively). **f, g** Same as **d, e** for uIPSCs (*n* = 19 and 12, respectively). No significant differences were found in the amplitude and area of uEPSCs and uIPSCs across L2/3 and L5BL pyramidal neurons. **h** Stimulus intensity (left panel) and latency (right panel) of unitary IPSCs vs EPSCs. Only neurons in which both responses were recorded were considered (*n* = 11 L2/3 and 7 L5BL pyramidal neurons, gray and black circles, respectively). Notice that, as expected, uIPSCs require a higher stimulation threshold and appear at longer latencies than uEPSCs

To better understand the synaptic basis of the different sizes of the PSPs recorded in L2/3 and L5BL pyramidal neurons, we studied the properties of the excitatory and inhibitory postsynaptic currents (EPSCs and IPSCs) evoked by contralateral stimuli. First, we studied the unitary EPSCs and IPSCs (uEPSCs and uIPSCs) in a non-paired sample of L2/3 and L5BL pyramidal neurons with a minimal stimulation protocol (see “Methods”). In Fig. 2a, we show an example of a putative uIPSC (blue traces) and a putative uEPSC (red traces) recorded in the same pyramidal neuron. Notice the large number of failures and the stability of their amplitude across successful trials, suggesting their single-axon nature. Importantly, the amplitude and area of the uEPSCs and uIPSCs were not significantly different across both pyramidal cell subtypes (Fig. 2d, e for uEPSCs and f, g for uIPSCs, respectively). Also, and according to their disynaptic nature, unitary IPSCs had longer latencies and higher threshold than uEPSCs (Fig. 2a, h).

Next, we studied the compound EPSCs and IPSCs (cEPSCs and cIPSCs) in a paired sample of L2/3 and L5BL pyramidal cells that were patched sequentially. Within a pair, the stimulus electrode position was not changed from the first to the second neuron and the stimulus intensities used were the same; therefore, we assumed that the pool of neurons recruited by the stimulus was the same for both neurons in a given pair. Additionally, special attention was paid in each pair to select neurons whose apical dendrites were radially aligned. In these pairs, we used intermediate (233 ± 20 µA, *n* = 12 pairs, 6 slices from 6 mice) and near-maximal stimulus intensities (571 ± 51 µA, *n* = 14 pairs, eight slices from eight mice; in nine of these pairs, both intensities were tested). Notice that both stimulus intensities were larger than those employed to evoked unitary currents.

**Fig. 3:**
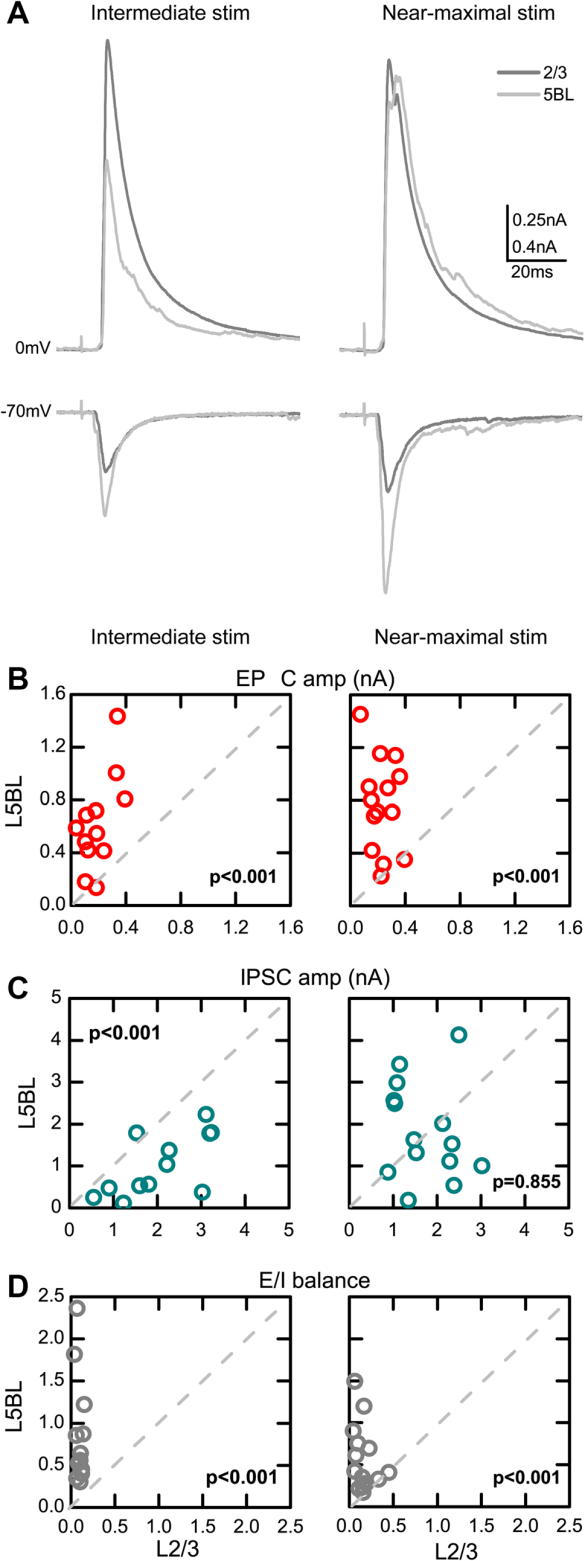
Larger excitation/inhibition balance in the cortical response of L5BL vs L2/3 pyramidal neurons. **a** Example of the compound EPSCs and IPSCs evoked in a L2/3 (dark gray) and a L5BL pyramidal neuron (light gray). Traces represent the average of ten successive responses to a 200 µA (left panel) and 500 µA stimulus pulse (right panel). Notice that the compound EPSC is larger in the L5BL pyramidal neuron at both intensities tested, while the compound IPSC is larger in the L2/3 pyramidal neuron in response to 200 but not to 500 µA stimulation. **b**–**d** Peak amplitude of the compound EPSCs (**b**), compound IPSCs (**b**), and excitation to inhibition balance (**d**, E/I ratio as cEPSC/cIPSC amplitude) for a paired sample of L2/3 and L5BL pyramidal neurons. Each dot represents the values for the responses recorded in both neurons from a pair. Data in **b–d** were obtained in response to intermediate stimulus intensities (left panels, range 150–400 µA; mean ± SEM: 233 ± 20 µA, *n* = 12 pairs, six slices from six mice) and in response to near-maximal stimulus intensities (right panels, range 500–800 µA; mean ± SEM: 571 ± 51 µA, *n* = 14 pairs, eight slices from eight mice). In nine pairs, both intensities were tested. Notice that the E/I balance is more favorable to excitation in L5BL pyramidal neurons at both intensities tested

Examples of cEPSCs and cIPSCs are shown in Fig. 3a. With intermediate stimulus intensities, evoked EPSCs were significantly smaller in L2/3 than in L5BL neurons, while IPSCs were significantly larger in L2/3 than in L5BL pyramidal cells (left panels in Fig. 3a–c). With near-maximal stimuli, EPSCs remained significantly smaller in L2/3 neurons, while no significant differences were found in IPSC size (right panels in Fig. 3a–c). Similar results were obtained when the area of the postsynaptic current, instead of the peak amplitude, was considered (data not shown). Overall, the excitatory to inhibitory balance of the cortical response (E/I balance), measured as the cEPSC to cIPSC peak amplitude ratio, was significantly more favorable to excitation in L5BL pyramidal neurons at both stimulus intensities (Fig. 3d).

**Fig. 4:**
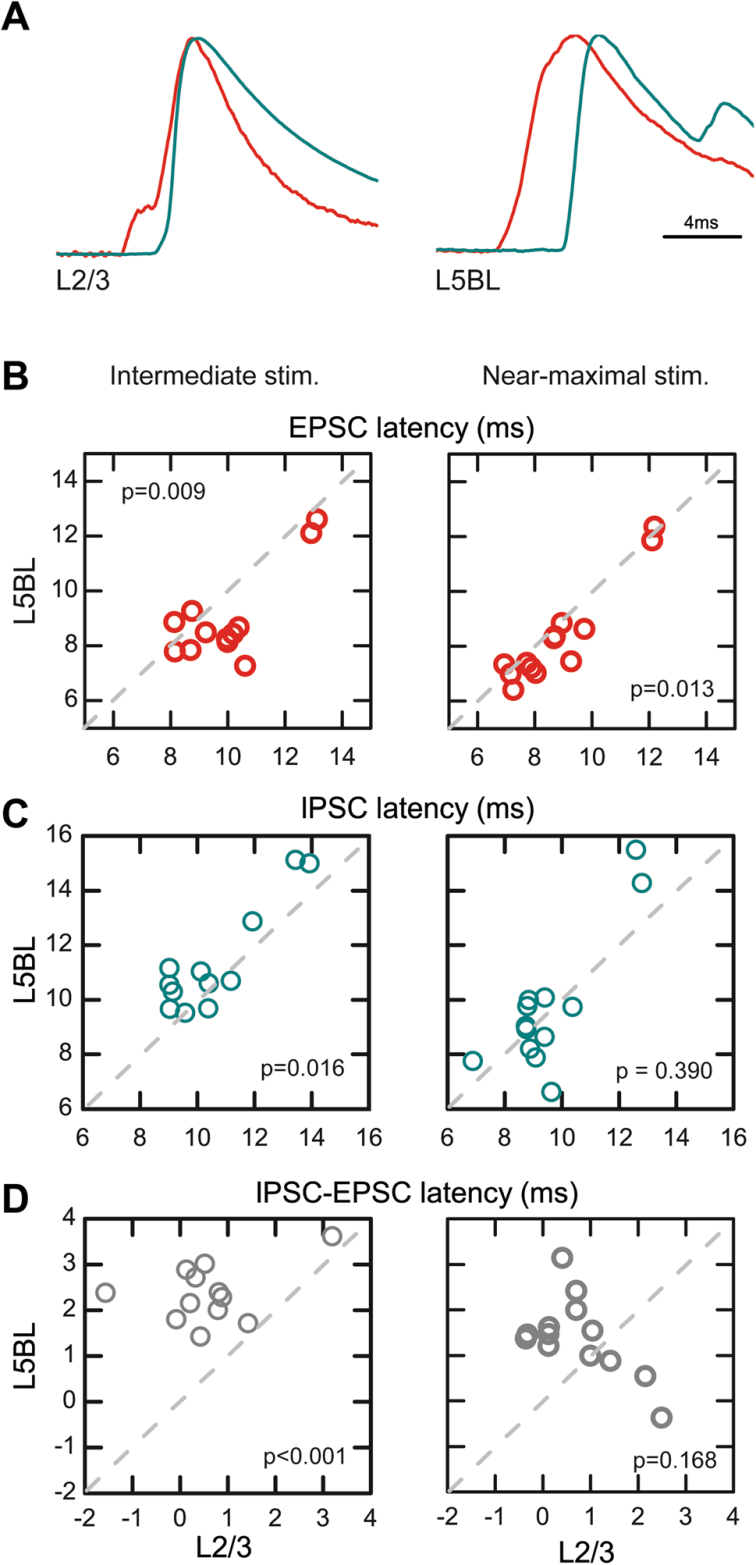
Longer time window to integrate excitation in L5BL vs L2/3 pyramidal neurons. **a** Example of the compound EPSCs (red) and IPSCs (blue) evoked in a pair formed by a L2/3 and a L5BL pyramidal neurons sequentially recorded. Stimulus intensity: 200 µA. Each trace represents the average of ten consecutive responses normalized to peak amplitude. cEPSCs have been inverted to facilitate comparison with cIPSCs. **b–d** Latency of the cEPSC (**b**), cIPSC (**c**) and cIPSC–cEPSC latency (**d**) in a paired sample of L2/3 and L5BL pyramidal neurons (same as in Fig. 4). Notice that with intermediate (**b–d**, left panels) but not with near-maximal stimulus intensities (**b–d**, right panels), there is a significantly longer time window to integrate excitatory input before the arrival of inhibition in L5BL vs L2/3 pyramidal neurons

In addition to the different size of the cEPSCs and cIPSCs, we also found differences in the latency of both components (Fig. 4; the latency was measured at the 50% of the rising phase). With intermediate intensities, L5BL pyramidal cells had cEPSCs of significantly shorter latency and cIPSCs with significantly longer latency than L2/3 pyramidal cells, giving rise to a longer time window to integrate excitatory inputs before the arrival of inhibition in L5BL cells (see examples in Fig. 4a and quantification in 4B–D, panels in the left). With near-maximal stimuli, the difference in E–I time window between both pyramidal subtypes disappeared (Fig. 4b–d, panels in the right). Therefore, the responsiveness of L5BL pyramidal neurons was favored with respect to those in layer 2/3 by a larger E/I balance and, in the response to intermediate stimulation range, by a longer time window to integrate excitation before the arrival of inhibition. The recordings of synaptic currents were made using an intracellular solution based on Cs-methanesulfonate (see “Methods”) to increase the membrane resistance and to improve the clamping of the membrane; however, we cannot totally rule out the possibility that the comparison of synaptic currents in two types of pyramidal neurons with dendritic trees of different sizes could introduce a bias in the sizes of the synaptic currents.

### Stronger recruitment of layer 2/3 vs layer 5 PV-FS interneurons by cortical inputs

**Fig. 5:**
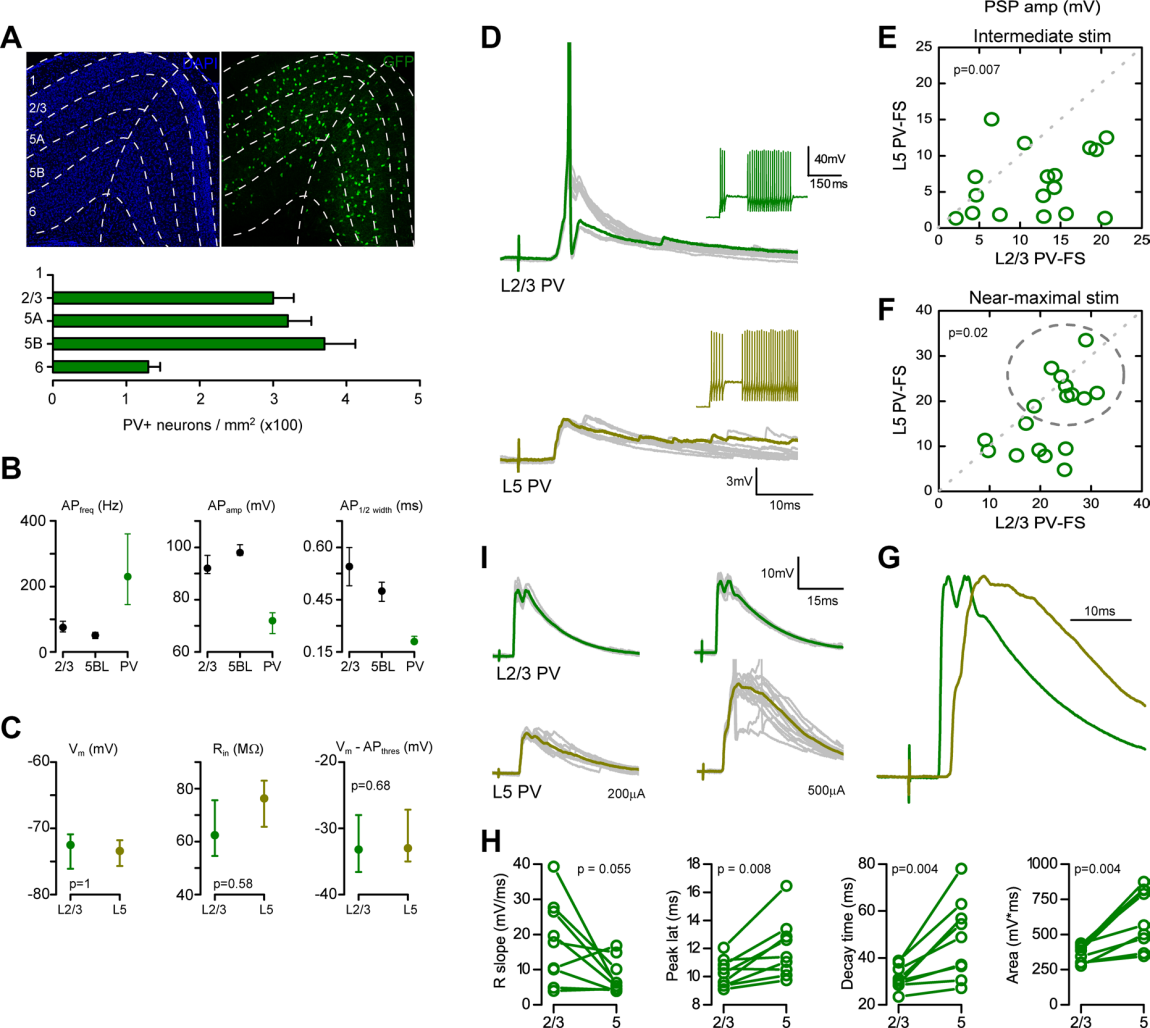
PV-FS interneurons in layer 5 are less excitable than PV-FS cells in layer 2/3. **a** Quantification of PV + somas across layers in the retrosplenial agranular cortex by immunostaining with an anti-PV antibody in 40-µm slices (*n* = 6 slices from 3 brains; an example is shown in the upper panel in the right). Laminar boundaries were established according to DAPI staining (left panel). Notice that PV + soma density was similar across layers 2/3, 5A and 5B. **b** EGFP + neurons from Pvalb-Cre;RCE mice slices were clearly distinguishable from pyramidal cells according to their firing frequency, AP amplitude and AP half width in response to depolarizing somatic current steps (575 pA for firing frequency quantification). **c** No difference were detected in the resting potential, input resistance and resting to action potential threshold difference in a paired sample of L2/3 and L5 PV-FS interneurons (*n* = 17 pairs sequentially recorded, 11 slices from 10 mice). Within this sample, the soma position in the cortical column was (in µm from pia, median and range): L2/3 PV-FS: 210, 150–340 µm; L5 PV-FS: 510, 400–650 µm. **d** Example of the postsynaptic potentials evoked in one of such pairs (upper panel L2/3 PV-FS; lower panel L5 PV-FS interneuron; stimulus intensity 500 µA). 10 successive responses are shown, one highlighted in green. Notice that the layer 2/3 PV-FS interneuron, but not the one in layer 5, fires in response to the stimuli (AP truncated). The insets in the left show the firing pattern of both neurons in response to the intracellular injection of suprathreshold current steps. **e, f** PSP peak amplitude in the same sample of L2/3 and L5 PV-FS interneurons shown in panel **c**. Two stimulus intensities were employed in each pair (in µA, mean ± SEM): 165 ± 50 µA (**e**) and 483 ± 71 µA (**f**). Notice that PSP peak amplitude was larger in layer 2/3 PV-FS interneurons at both intensities tested. Each dot represents the peak amplitude of the PSPs recorded for both neurons in a pair. **g** Example of a L2/3 vs L5 PV-FS pair in which the AP threshold was reached in some trials in the deeper neuron (AP truncated). Notice that for the superficial PV-FS cell, the responses at both intensities tested are similar, while for the deep PV-FS cell, the response at 200 µA is smaller, but increases with the 500 µA stimulus. **h** Normalized subthreshold PSPs evoked in the pair of PV-FS cells shown in **d** (stimulus intensity 500 µA). Notice the different time course of both responses. **i**. PSP rise slope (20–80%), peak latency, decay time (from peak to 10% of PSP amplitude) and area for the nine pairs that appear within the dotted line in **f**

We have previously shown that, in our conditions, inhibitory currents evoked in pyramidal neurons are largely dependent on the recruitment of PV-FS interneurons from layers 2–5, while non-PV-FS interneurons rarely fired in response to single-pulse contralateral stimulation (Sempere-Ferràndez et al. 2018). Quantification of cell somas immunoreactive to an anti-PV antibody revealed that in the retrosplenial cortex, PV-FS interneuron density was similar across layers 2/3, 5A and 5B, suggesting that the smaller size of the IPSCs in layer 5BL pyramidal neurons was not due to a reduction in the number of PV-FS interneurons in deeper layers (Fig. 5a). Thus, we hypothesized that the difference in the properties of the compound IPSCs between L2/3 and L5BL pyramidal neurons could be explained if superficial PV-FS cells were more responsive to contralateral stimulation than those of layer 5. To assess this possibility, we compared the postsynaptic responses evoked in PV-FS interneurons of both layers following the same stimulation protocol than in Fig. 1. During recordings, PV-FS interneurons were identified as GFP + cells in brain slices made from Pvalb-Cre;RCE mice. Additionally, they were reliably distinguished from pyramidal neurons by their high-frequency firing pattern in response to 575 pA current steps and by the action potential shape (measured by its amplitude and the half-width duration (Fig. 5b).

In a paired sample of L2/3 and L5 PV-FS, interneuron-evoked PSPs were significantly larger in layer 2/3 PV-FS cells in response to intermediate (165 ± 50 µA) and near-maximal (483 ± 71 µA) stimulus intensities (*n* = 17 pairs of sequentially recorded neurons). An example of responses evoked with near-maximal stimulation in a pair of PV-FS interneurons is shown in Fig. 5d (for quantification see Fig. 5e, f). With intermediate stimuli, 2 out of 17 layer 2/3 PV-FS cells and 0 out of 17 layer 5 PV-FS neurons fired (*p* = 0.144). With near-maximal stimuli, eight out of 17 layer 2/3 and two out of 17 layer 5 PV-FS neurons fired (*p* = 0.024). Importantly, no significant differences were detected between PV-FS neurons of layer 2/3 and layer 5B in resting membrane potential, membrane input resistance and in the difference between resting and threshold potential (Fig. 5c), indicating that the weaker responsiveness of layer 5 PV-FS cells depended on a weaker synaptic drive and not on a lower intrinsic excitability.

In spite of the larger amplitude of the PSPs evoked in layer 2/3 with respect to layer 5 PV-FS cells, in some layer 5 PV-FS neurons, near-maximal stimuli evoked large PSPs (> 15 mV; neurons shown within a dotted circle in Fig. 5f). An example of a PV-FS pair including one deep PV-FS interneuron that reached the AP threshold in response to a 500 µA pulse is shown in Fig. 5j. In this subset of PV-FS neurons, PSP amplitude was similar in the superficial and in the deep PV-FS neurons (PSP amplitude in mV: L2/3 PV-FS median 25.5, range 18.9–31.3; L5 PV-FS median 21.8, range 18.8–33.5; *p* = 0.25) but the time course of the PSPs was clearly different: the rising and decay phases of the PSPs were slower and the peak was delayed in layer 5 PV-FS neurons (Fig. 5i). As a consequence, the total PSP area was significantly larger in L5 PV-FS neurons.

### Different recruitment of PV-FS and pyramidal neurons across layers

**Fig. 6:**
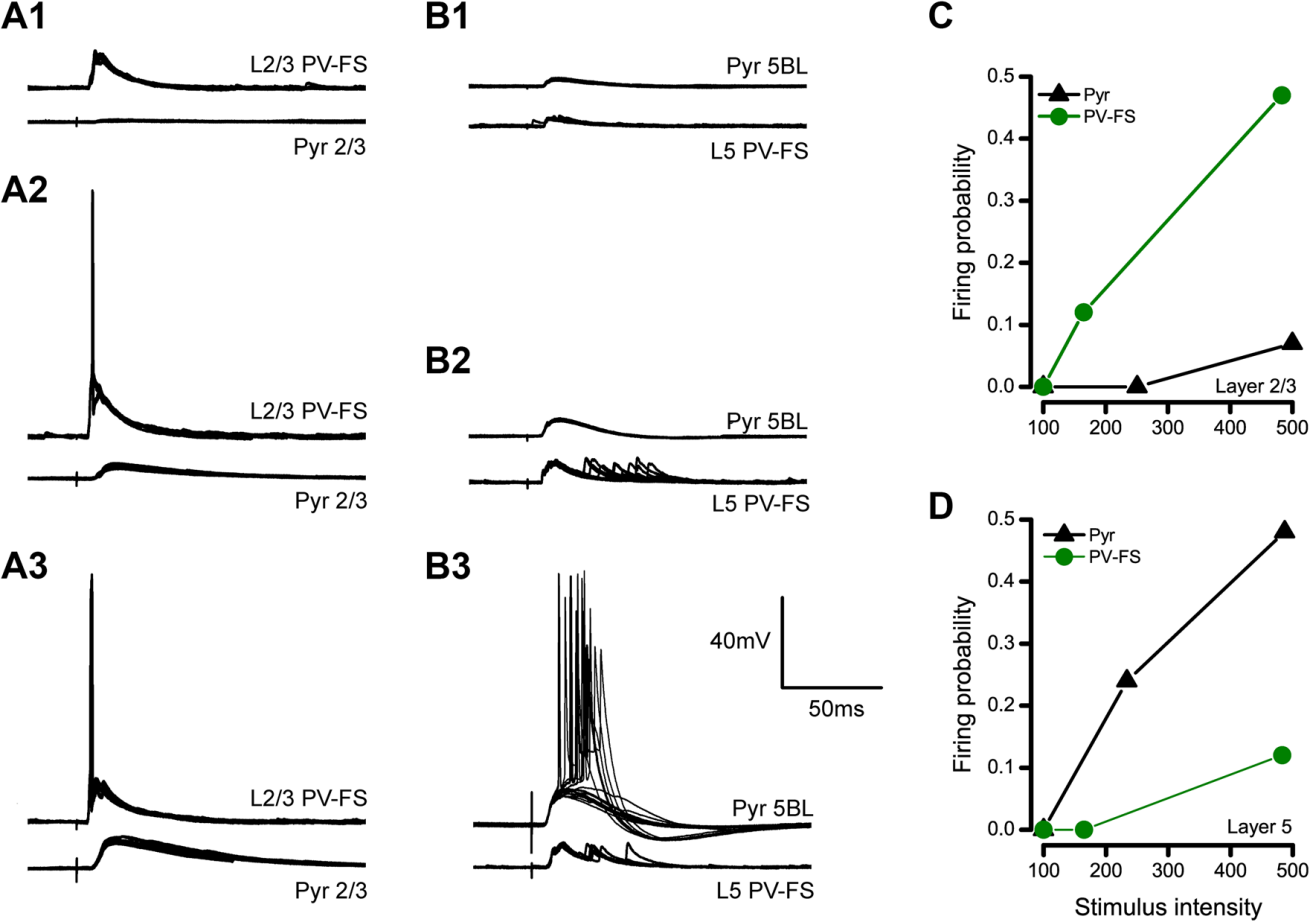
Opposite sequence in the recruitment of pyramidal and PV-FS neurons in layers 2/3 and 5. **a** PSPs evoked in a pair of recorded neurons formed by a pyramidal cell and a PV-FS interneuron in layer 2/3 in response to 100 (**a**1), 200 (**a**2) and 500 µA stimulation (**a**3). 10 consecutive responses are shown superimposed in each case. Notice that the PV-FS interneuron fires in response to 200 and 500 µA pulses, while the responses are subthreshold in the pyramidal neuron. **b** Same as in **a**, but in this case for a pair of neurons formed by a L5BL pyramidal neuron and a layer 5 PV-FS interneuron. Notice that the L5BL pyramidal neuron fires in response to 500 µA pulses while the responses are subthreshold in the PV-FS cell. Also notice the presence of long latency and large jitter PSPs in the PV-FS cell. **c, d** Firing probability for pyramidal and PV-FS interneurons from layer 2/3 (**c**) and 5 (**d**) with respect to stimulus intensity

We have combined the data related to the firing of pyramidal and PV-FS interneurons in response to contralateral stimulation in Fig. 6 to compare the recruitment of these neurons across layers (the data on pyramidal neurons is the whole sample of neurons, which includes the paired recorded neurons shown in Fig. 1 and the no-paired recordings). Overall, our data show that pyramidal neurons and PV-FS interneurons were recruited in a different sequence across layers by stimuli of increasing strength: in layer 2/3, PV-FS interneurons were recruited with a lower stimulus threshold than pyramidal neurons (Fig. 6a, c), while the opposite situation occurred in layer 5 (Fig. 6b, d).

Several reports indicate that large, thick-tufted pyramidal neurons in layer 5B make excitatory synapses on layer 5 PV-FS interneurons (Angulo et al. 2003; Lourenço et al. 2014). This observation, in combination with the fact that these neurons have a lower firing threshold than layer 5 PV-FS interneurons, suggests that PV-FS cells receive excitatory input from L5BL pyramidal neurons before reaching their firing threshold. This could explain the larger area and prolonged time course of the PSPs evoked in layer 5 PV-FS interneurons with respect to those in superficial layers.

## Discussion

### Differences in the responsiveness of L2/3 and L5BL pyramidal neurons depend on synaptic mechanisms

We have shown that, in slices, stimulation of superficial layers of the cortex often recruited L5BL pyramidal neurons in the contralateral hemisphere, but mostly evoked subthreshold PSPs in L2/3 pyramidal neurons (Fig. 1). This resembles the spontaneous and evoked output observed in both pyramidal neuron subtypes during in vivo experiments in anesthetized and awake animals (Beloozerova et al. 2003a, b; de Kock et al. 2007; Greenberg et al. 2008; de Kock and Sakmann 2009), prompting us to use our preparation to investigate the underlying mechanisms of such difference.

Here we demonstrate that the different recruitment in response to excitatory inputs between both pyramidal cell types depends on synaptic mechanisms. In L5BL pyramidal neurons, the amplitude and area of compound EPSCs evoked by cortico-cortical inputs were larger with respect to those of L2/3 pyramidal neurons in response to a wide range of stimulus intensities (Fig. 3). Additionally, IPSCs were delayed (Fig. 4) and their stimulus–response relationship was shifted to stronger stimuli in L5BL neurons with respect to L2/3 ones (Fig. 3).

Our results were obtained from 17- to 21-day-old mice; at this age the postnatal development of the cortical circuit is still not completed and, therefore, we cannot rule out the possibility that further circuit refinement may participate in the mechanisms controlling pyramidal neurons firing and the different coding strategies of layer 2/3 and layer 5 pyramidal neurons. As an example, Angulo et al. (1999a) showed that from 3 to 5 postnatal weeks some changes occur in the layer 5 pyramidal neurons to FS interneuron excitatory connections, particularly a switch from paired-pulse depression to paired-pulse facilitation that confers layer 5 pyramidal neurons wider integrative capabilities at 5 weeks of postnatal age. This switch to facilitation of the pyramidal to FS pathway could increase the firing of FS neurons induced by layer 5 pyramidal neurons and, therefore, could reinforce the inhibitory feedback inhibition on pyramidal neurons.

### Possible origin of the larger size of the compound EPSCs in L5BL pyramidal neurons

As mentioned before, we found that a major determinant of the larger recruitment of L5BL with respect to L2/3 pyramidal neurons was the larger size of the compound EPSCs in the formers (Fig. 3). In turn, this could depend on differences in the properties of unitary connections and/or in the degree of convergence of cortical excitatory inputs on L2/3 and L5BL pyramidal neurons, but our results do not clarify this issue.

In our experiments, there are three sources of excitatory inputs. First, a major source is the callosal projecting neurons located contralaterally to the recording region that are directly recruited by the electrical pulse. Using a minimal stimulation protocol, we have shown that putative unitary EPSCs evoked in layer 2/3 and layer 5B large pyramidal neurons had a similar amplitude and area in both pyramidal cell subtypes (Fig. 2). In these conditions (stimulus intensities 50–100 µA), excitatory synaptic responses are mostly driven by these callosal projecting neurons, as pyramidal neurons ipsilateral to the recording site require higher stimulus intensities to fire (Sempere-Ferràndez et al. 2018). This suggests that the size of unitary callosal synapses is not contributing to the larger compound EPSCs in L5BL pyramidal neurons. Second, L5BL pyramidal neurons are often recruited by the stimuli (Figs. 1, 5 out of 21 and 10 out of 21 L5BL pyramidal cells fired in response to 200 and 500 µA stimuli); therefore, they are a relevant source of excitatory input. In the primary sensory cortex of the rodent brain, L5BL pyramidal neurons are connected with other L5BL pyramidal cells with a probability of about 0.1 (Markram et al. 1997; Song et al. 2005). However, their inputs on L2/3 pyramidal neurons are scarce (Reyes and Sakman 1999; Lefort et al. 2009), indicating that in our conditions, recurrent connectivity in layer 5 must amplify the postsynaptic responses in L5BL, but not in L2/3 pyramidal neurons, partially explaining the larger size of the compound EPSC in these neurons. Third, L2/3 pyramidal neurons ipsilateral to the recording site constitute a minor source of excitatory input, at least in response to high-intensity stimuli (Fig. 2 out of 28 L2/3 pyramidal cells fired in response to 500 µA stimuli). In several cortical regions, superficial pyramidal neurons have been shown to synapse with other L2/3 but also with L5BL pyramidal neurons. Importantly, the connection probability is higher in the L2/3 to L5BL pathway (about 0.25; Thomson and Bannister 1998) than in the L2/3 to L2/3 pathway (0.1–0.15; Holmgren et al. 2003; Avermann et al. 2012).

Recently, it has been described that the molecular mechanism involved in the formation of intracolumnar L2/3–L5BL pyramidal neurons also applies for the formation of callosal synapses with contralateral L5BL pyramidal cells (Harwell et al. 2012). It is then likely that a combination of (1) a higher convergence of callosal axons (and axons from ipsilateral L2/3 pyramidal neurons) on L5BL vs L2/3 pyramidal neurons with (2) the recurrent connectivity in layer 5B, may explain the larger size of the EPSCs of L5BL with respect to L2/3 pyramidal neurons.

### Lower recruitment of layer 5 PV-FS cells as a main contributor to the stronger responsiveness of L5BL pyramidal neurons

The smaller size and longer latency of the compound IPSCs evoked in L5BL with respect to L2/3 pyramidal neurons (Figs. 3, 4) are another major determinant of the larger responsiveness of the L5BL neurons to cortico-cortical inputs. Given the similarity in the properties of the unitary IPSCs among both pyramidal neuron subtypes (Fig. 2), including their peak amplitude and area, the observed differences in the properties of the compound IPSCs suggested that different sets of inhibitory neurons were responsible for the inhibition in both neuron types.

In our preparation, PV-FS interneurons from superficial layers responded with PSPs of larger amplitude and were more often recruited by the stimulus than PV-FS cells in layer 5 (Fig. 5). We show that the intrinsic electrophysiological properties of PV-FS cells, particularly their membrane input resistance, resting potential and AP threshold were not laminar dependent, suggesting that the difference in the response amplitude between PV-FS neurons in layers 2/3 and 5 was caused by synaptic mechanisms. This result is in line with the recent description of the role of the transcription factor Er81 in the synaptic excitability of PV-FS cells (Dehorter et al. 2015). Higher expression levels of Er81 protein cause a higher E/I balance in these interneurons. In line with our results, Er81 is more abundant among superficial PV-FS cells. Thus, we propose that the lower excitability of layer 5 PV-FS cells, with respect to those in superficial layers, contributes to the lower amplitude of the IPSCs in L5BL pyramidal neurons and, as a consequence, to their larger responsiveness to incoming excitatory inputs with respect to pyramidal neurons in superficial layers.

The plausibility of this hypothesis is reinforced by several considerations. First, we have previously shown that in our conditions (single-pulse stimulation of the contralateral cortex in vitro), PV-FS interneurons are recruited with a higher probability than non-PV-FS interneurons, suggesting that they are the main contributors of the inhibitory currents evoked in pyramidal neurons (Sempere-Ferràndez et al. 2018). This is in agreement with published data indicating that PV-FS interneurons are more responsive to single-pulse stimulation in cortical (Holmgren et al. 2003; Mateo et al. 2011; Avermann et al. 2012) and thalamocortical preparations (Gabernet et al. 2005; Cruikshank et al. 2007). However, with this argument we do not intend to exclude the possible role of other types of interneurons. It is likely that, in addition to PV-FS interneurons, non-PV-FS interneurons are also contributing to the IPSCs recorded in the pyramidal neurons included in this study. Indeed, it has been documented that burst firing in L5BL pyramidal neurons, similar to what we show in Fig. 1, can recruit somatostatin-positive interneurons, which in turn provide disynaptic inhibition to other L5BL pyramidal cells (Silberberg and Markram 2007). Future experiments should address the laminar contribution of somatostatin interneurons to the inhibitory cortical subnetworks.

Second, studies of cortical connectivity indicate that inhibition in pyramidal neurons is dominated by homolaminar connections, that is, most of the inhibitory synapses in a given pyramidal neuron arise from interneurons whose somas are located in the same layer (Kätzel et al. 2011). Unfortunately, a detailed analysis of the axonal arborizations of PV-FS interneurons in the retrosplenial cortex is missing, but in other agranular territories, such as the motor cortex, the axons of PV-FS interneurons mostly arborize within the limits of their layer (Tanaka et al. 2011). Although a subpopulation of PV-FS cells with interlaminar projections exist (Bortone et al. 2014), these neurons have been described in primary visual cortex, mostly in layer 6, and they are implicated in thalamocortical loops. However, in response to our stimulating protocol, layer 6 PV-FS interneurons remain unresponsive and the synaptic sources activated are intracortical and not thalamic (Sempere-Ferràndez et al. 2018).

Third, it can be hypothesized that the lower amplitude of the cIPSCs on L5BL pyramidal neurons could be explained by a reduced PV-FS to L5BL vs L2/3 connectivity, which in turn could arise from a lower abundance of PV-FS cells or a lower PV-FS to pyramidal connection probability in layer 5 vs 2/3. However, there are data against both possibilities. We have quantified the amount of PV-FS cells across layers of the retrosplenial cortex and our data indicate that if any difference, PV-FS cells are more abundant in layer 5 (Fig. 5a); similarly, no differences have been found in the PV-FS to pyramidal connection probability across layers (Packer and Yuste 2011). Therefore, it seems reasonable to think that the different responsiveness of PV-FS cells across layers is the cause of the lower amplitude of the IPSCs in layer 5BL pyramidal neurons.

### Feed-forward vs feedback inhibition

Our data also suggest that the recruitment of pyramidal and PV-FS cells followed different sequence in response to stimuli of increasing intensity across layers. In superficial layers, PV-FS cells had a lower stimulation threshold to fire than pyramidal neurons (Fig. 6). This is in agreement with several studies that have investigated the role of PV-FS interneurons in the superficial layers of the cortex. In vitro and in vivo, bulk stimulation of superficial pyramidal neurons or thalamocortical projecting neurons resulted in a potent recruitment of PV-FS cells and the silencing of the majority of the pyramidal neuron population in their target territories (Avermann et al. 2012; Cruikshank et al. 2007; Gabernet et al. 2005 and; Mateo et al. 2011). In both cases, PV-FS cells were recruited more abundantly and with a lower stimulus threshold than surrounding pyramidal neurons. Three main factors, the larger convergence of excitatory inputs and the larger amplitude of the unitary EPSCs in PV-FS cells than in pyramidal neurons, in combination with the large divergence of PV-FS to pyramidal neuron connectivity, explain these observations (Avermann et al. 2012; Cruikshank et al. 2007; Gabernet et al. 2005; Mateo et al. 2011). Therefore, in superficial layers, PV-FS cells establish a potent feed-forward inhibitory control over cortical responsiveness.

In contrast, in layer 5, we observed that L5BL pyramidal cells were recruited with lower stimulus intensities than PV-FS cells (Fig. 6b, d). We propose that the requirement of higher stimulation intensities to evoke large amplitude PSPs and, importantly, the shorter time course of the responses in layer 5 PV-FS interneurons compared to PV-FS cells in superficial layers (Fig. 5), depended on the fact that in deeper layers, PV-FS cells compensate their low responsiveness to the monosynaptic excitatory input evoked by the stimulus with the disynaptic input from surrounding L5BL pyramidal neurons. This seems reasonable in the light of the following considerations: (1) several reports demonstrate that L5BL neurons are synaptically connected with L5 PV-FS interneurons (Angulo et al. 1999b, 2003; Lourenço et al. 2014), (2) our results show a strong recruitment of L5BL pyramidal neurons by contralateral stimulation and (3) according to their latency, the action potentials evoked in L5BL pyramidal neurons can explain the delayed time course of the PSPs of layer 5 PV-FS interneurons (compare Fig. 1c–e with Fig. 5h–i). Altogether, this suggests that in contrast to what happens in superficial layers, PV-FS interneurons in layer 5 constitute a feedback inhibitory control of pyramidal neuron responsiveness.

Recently, it has been shown that inhibitory synapses from L5 PV-FS cells on L5BL pyramidal neurons, but not from somatostatin-positive (SOM) interneurons, are potentiated in response to postsynaptic firing in a non-associative manner (Lourenço et al. 2014), that is, without requiring the firing of the PV-FS cell. This form of plasticity is not cellular specific, as the activation of a single L5BL pyramidal neuron can potentiate the PV-FS synapses on surrounding pyramidal cells. These imply that PV-FS-dependent inhibition strongly depends on the previous firing of surrounding L5BL pyramidal neurons, stressing their role as feedback inhibitors.

Our experiments were done studying the activation of the local cortical circuit of layers 2/3 and 5B by cortico-cortical axons originated in the contralateral hemisphere, and we believe that our conclusions may be generalized to the responses evoked by ipsilateral cortico-cortical axons (Adesnik and Scanziani 2010; Mateo et al. 2011; Lourenço et al. 2014). However, the question of whether the synaptic mechanisms controlling the different firing of layer 2/3 and layer 5B pyramidal neurons that we propose could be extended to extra-cortical inputs remains open. It is likely that the same mechanisms, at least in part, would apply in response to extra-cortical inputs since the different firing code observed in these two groups of cortical neurons has been observed in different functional conditions and in different cortical areas and, therefore, it should be at least a part of the general principles governing the response of cortical microcircuits to incoming inputs.

## Electronic supplementary material

Below is the link to the electronic supplementary material.

Supplementary material 1 (DOCX 273 KB)
