# Supplementary material for: Synaptic mechanisms underlying the intense firing of neocortical layer 5B pyramidal neurons in response to cortico-cortical inputs
Source: Brain Struct Funct. 2019 Feb 12;224(4):1403–16. doi: 10.1007/s00429-019-01842-8 (PMC6509071; doi:10.1007/s00429-019-01842-8)
Supplement: Supplementary file 1 — Supplementary material 1 (DOCX 273 KB) [file 429_2019_1842_MOESM1_ESM.docx]

**
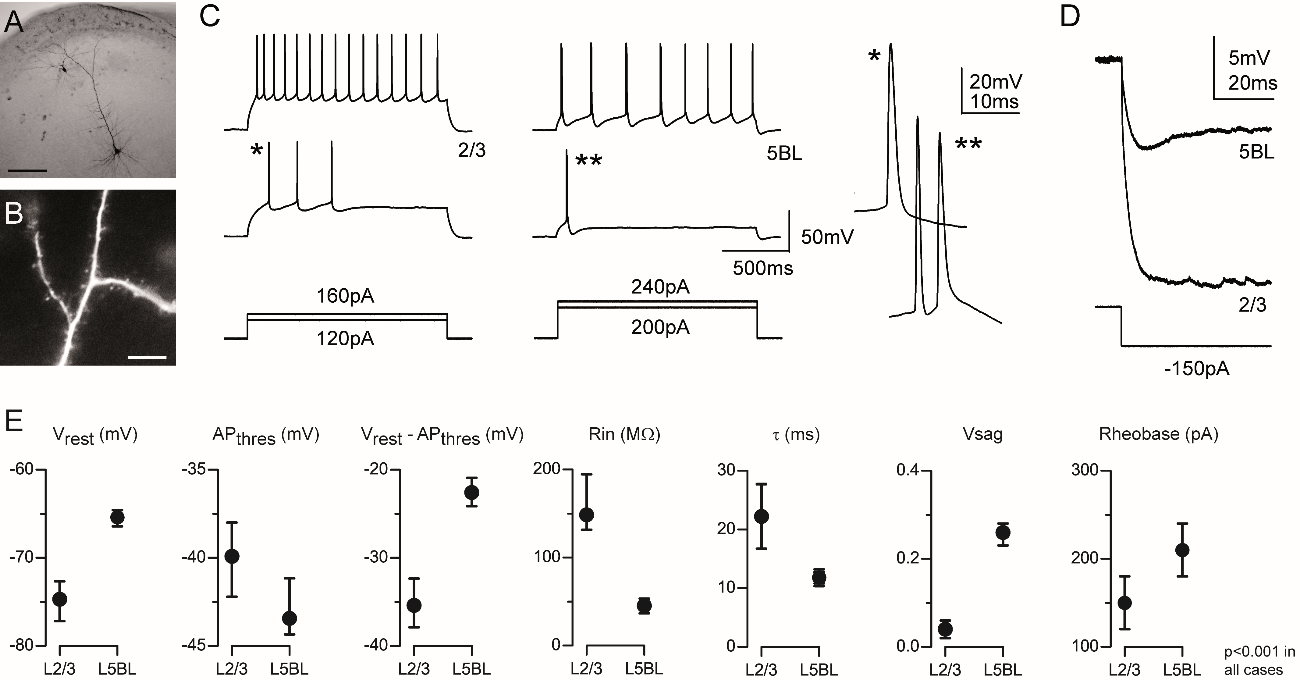
**

**Supplementary figure 1. Intrinsic electrophysiological properties of L2/3 and L5BL pyramidal neurons.**

*A,* morphological reconstruction of a L2/3 and a L5BL pyramidal neurons after intracellular perfusion with biocytin. Notice the large apical tuft of the L5BL pyramidal cell in layer 1. *B,* detail of the apical dendrite of a L2/3 pyramidal neuron intracellularly perfused with a solution containing the fluorescent dye Alexa Fluor 594. Notice the abundant presence of dendritic spines. *C-D*, voltage responses from the neurons shown in A to intracellular injection of square current steps. Notice the larger rheobase of the L5BL pyramidal neuron. The initial spiking response of the L5BL pyramidal neuron was a doublet of action potentials (right panel), while a single spike appeared in the L2/3 pyramidal neuron. In D, the response to a hyperpolarizing current step is shown superimposed for both neurons. Notice the smaller amplitude and larger voltage sag of the response in the L5BL pyramidal cell. *E*, summary of the intrinsic electrophysiological properties of a sample of L2/3 and L5BL pyramidal neurons (n = 28 and 21, respectively). Data is shown as the median and the quartile 1 – quartile 3 range; p < 0.001 for all cases.
